# Severe cutaneous toxicity following treatment with radiotherapy and cetuximab: a case report

**DOI:** 10.1186/1757-1626-2-25

**Published:** 2009-01-07

**Authors:** Arun Azad

**Affiliations:** 1 Ludwig Institute for Cancer Research, University of Melbourne, Austin Hospital, Austin Health, 145-163 Studley Road, Heidelberg, Victoria, 3084, Australia

## Abstract

While the addition of cetuximab to radiotherapy improves clinical outcomes in locoregionally advanced head and neck squamous cell cancers, there are a small number of reports of severe radiation dermatitis occurring with this therapeutic combination. We present the case of a 69 year old male who developed severe radiation dermatitis following treatment with cetuximab and radiotherapy for a locoregionally advanced head and neck squamous cell cancer.

## Introduction

The addition of cetuximab to radiotherapy improves local control and overall survival in locoregionally advanced head and neck squamous cell cancer. However, evidence is emerging that severe radiation dermatitis can occur in patients receiving cetuximab. We present the case of a 69 year old male who developed severe radiation dermatitis following treatment with cetuximab and radiotherapy for a locoregionally advanced head and neck squamous cell cancer.

## Case presentation

A 69-year-old Caucasian male developed a severe rash two weeks after starting treatment with concurrent cetuximab and radiotherapy for a loco-regionally advanced right buccal head and neck squamous cell cancer (HNSCC). Two months earlier, magnetic resonance imaging (MRI) identified a 3.8 × 1.4 × 3.6 cm right buccal mass with a 1.7 cm left sided midcervical lymph node. There was no other disease on fluorodeoxyglucose positron emission tomography (FDG-PET). His disease was considered unresectable and cisplatin was contra-indicated because of sensori-neural industrial hearing impairment. He commenced radiotherapy (66 Grays in 33 fractions) and cetuximab (250 mg/m^2 ^weekly during radiation, with a single dose of 400 mg/m^2 ^the week prior to radiation).

He had undergone resection of a lymph node-negative HNSCC of the left floor of mouth five years previously. In addition, a right retromolar trigone HNSCC had been resected 10 years previously. No post-operative radiotherapy was administered in either case. The only other history was hypertension treated with metoprolol. He had smoked 1 pack a day for 30 years, having ceased 30 years prior. He drank minimal alcohol. He lived with his wife and was a retired builder. There was no relevant family history.

Two weeks after commencing treatment he developed a severe painful skin reaction within the radiotherapy field. This progressively worsened, necessitating hospitalisation one week after completing radiotherapy. On examination, he had a moist, desquamating, circumferential, erythematous rash on the lower half of his face and upper neck. He was dehydrated and febrile at 38.5°C. He had oral mucositis but no candidiasis. There was a mild acneiform rash over the upper torso and arms. Other vital signs and the remaining physical examination were unremarkable.

Swabs were taken from the desquamating rash and cytomegalovirus DNA was detected on polymerase chain reaction. He was given a three-month course of valganciclovir (900 mg twice daily for three weeks, followed by 900 mg daily). The rash was treated with twice daily dressings and silver sulphadiazine cream and he was discharged home after three weeks. FDG-PET performed three months later showed complete metabolic remission.

Cetuximab is a chimeric monoclonal antibody against epidermal growth factor receptor (EGFR), a receptor tyrosine kinase that is up-regulated in more than 90% of HNSCCs[1]. In a pivotal phase III study of 424 patients with locoregionally advanced HNSCCs[2], the addition of cetuximab to radiotherapy significantly improved locoregional control (50% versus 41%), three-year survival (55% vs. 45%) and median survival (49 months versus 29 months). The only grade 3 toxicities more common in the cetuximab group were infusional reactions and acneiform rash (17% versus 1%).

Interestingly, there was no significant increase in grade 3 radiation dermatitis for the cetuximab group (23% versus 18%) in this study. However, multiple reports have subsequently emerged of severe radiation dermatitis complicating treatment with concurrent cetuximab and radiotherapy for HNSCCs [3-5]. Radiation dermatitis must be distinguished from the acneiform rash of the face, neck and upper torso that affects up to 2/3 of patients receiving anti-EGFR agents[6,7].

It should also be noted that most patients receiving radiotherapy alone for HNSCCs experience some degree of radiation dermatitis[8]. Nonetheless, consensus guidelines have now been developed for the prevention and management of cutaneous toxicity in patients with HNSCCs treated with cetuximab and radiotherapy[8]. These guidelines emphasise the need for adequate hygiene and hydration of the skin. More severe reactions may require dressings, topical anti-inflammatory agents and antimicrobials for secondary infections.

## Conclusion

The case presented here, in addition to others in the recent literature, highlights the risk of severe radiation dermatitis in patients with HNSCCs treated with cetuximab and radiotherapy. Clinicians must be aware of this risk, and implement appropriate strategies for prevention and treatment of radiation dermatitis in these patients.

## Consent

Written informed consent was obtained from the patient for publication of this case report and accompanying images. A copy of the written consent is available for review by the Editor-in-Chief of this journal.

## Competing interests

The author declares that they have no competing interests.

## Authors' contributions

AA was solely responsible for writing and editing this manuscript
